# Radioligands Targeting the Purinergic P2X Receptors

**DOI:** 10.3390/cells14130984

**Published:** 2025-06-27

**Authors:** Diego Dal Ben, Michela Buccioni, Catia Lambertucci, Beatrice Francucci, Aleksei Smirnov, Andrea Spinaci, Gabriella Marucci, Rosaria Volpini

**Affiliations:** School of Pharmacy, Medicinal Chemistry Unit, University of Camerino, Via Madonna delle Carceri, I-62032 Camerino, Italy; michela.buccioni@unicam.it (M.B.); catia.lambertucci@unicam.it (C.L.); beatrice.francucci@unicam.it (B.F.); aleksei.smirnov@unicam.it (A.S.); andrea.spinaci@unicam.it (A.S.); gabriella.marucci@unicam.it (G.M.); rosaria.volpini@unicam.it (R.V.)

**Keywords:** P2X receptors, radioligands, radiotracers, imaging, PET, single-photon emission computed tomography (SPECT)

## Abstract

Purinergic P2X receptors have a wide distribution within the body and modulate a number of physiological processes, being also involved in the development and progression of inflammation-, neuroinflammation-, neurodegeneration-, and cancer-related diseases. Radioligands that can detect specific P2X receptor subtypes and reveal their level of expression are of key importance for the development of novel P2X modulators, for the depiction of the involvement of these proteins in physio-pathological processes, and for the availability of novel diagnostic tools to be used for imaging experiments in vivo. Here we review and summarise the various P2X-targeting radioligands developed and reported to date, ranging from analogues of the endogenous P2X agonist ATP to the more recent and P2X subtype-selective allosteric modulators. Many of the high-affinity radioligands described are only suitable as in vitro receptor probes. No viable P2X3 or P2X4 radioligands for in vivo positron emission tomography (PET) imaging have been developed and reported to date. However, P2X7 antagonists, such as [^11^C]SMW139, [^11^C]GSK1482160, [^11^C]JNJ-54173717, and [^18^F]JNJ-64413739, have been successfully applied to PET imaging in the brain.

## 1. Introduction

**Figure 1:**
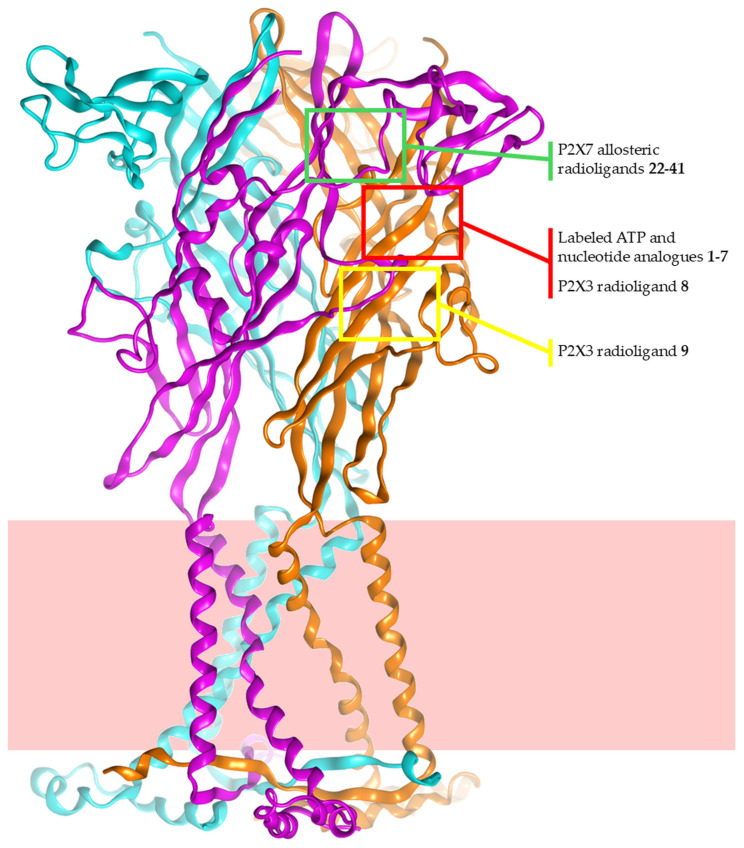
Location of binding sites for radioligands of the P2XRs. The depicted location is based on experimental 3D structures of P2XRs in complex with corresponding non-labelled analogues of compounds presented in this work, or from the experimental binding modes of structural analogues of the radioligands described. The red background is a schematic representation of the cell membrane.

The purinergic signalling system consists of purine nucleoside and nucleotide receptors, which are protein targets of the extracellular adenine nucleoside and nucleotide signalling activity. This general plot is modulated by the expression level of the specific receptor families and subtypes in the various districts of the body, as well as by the extracellular concentrations of the endogenous modulators. Extracellular ATP plays its signalling activity through the activation of the ionotropic P2X receptors (P2XRs, known as P2X1–7 subtypes). These proteins are homo- or hetero-trimers, with each monomer consisting of intracellular N- and C-termini, two transmembrane helix domains, and a large extracellular loop. The activation of P2XRs leads to the channel opening, resulting in the consequent flow of ions through the channel and altered cell transmembrane potential, as well as modulation of physio-pathological processes. A prolonged stimulation of the P2X7R subtype leads to the formation of a transmembrane pore allowing the passage of high-molecular-weight species [1]. The results of X-ray crystallography and cryo-electron microscopy experiments reported in recent years have provided a significant amount of P2XR structural data, which is of critical importance for the interpretation of mutagenesis studies and the rational design of modulators. These experiments have also provided a clear depiction of the location of both the orthosteric binding site, which is placed at the interface between two monomers in the extracellular region (hence a total of three ATP binding sites are present for each functional trimer), and of a number of additional binding sites for allosteric modulators for various P2XR subtypes (Figure 1).

In parallel to its signalling activity, ATP is degraded by the ectoenzyme CD39 to form ADP, whose following degradation by the ectoenzymes CD39 and CD73 leads to the nucleoside adenosine, which acts as a signalling molecule by the activation of the metabotropic G protein-coupled adenosine receptors (ARs, belonging to the rhodopsin-like family of GPCRs and consisting in the four subtypes: A_1_, A_2A_, A_2B_, and A_3_) [2]. While the extracellular ATP is considered a danger signal, extracellular adenosine acts as a “danger off” signalling molecule [3].

Furthermore, ATP, ADP, and further nucleotides like diadenosine polyphosphates and uridine nucleotides modulate the activity of the P2Y receptors. Even these ones belong to the rhodopsin-like family of GPCRs and consist of the P2Y_1,2,4,6,11,12,13,14_ subtypes, each one endowed with specific affinity and selectivity profiles for the above-cited nucleotides [4]. Diadenosine polyphosphates may also modulate the activity of P2XRs.

The widespread expression of the purinergic receptors, including the P2XRs, and their great importance in a large number of physiological processes and pathological conditions have made these proteins key targets for the development of pharmacological and therapeutic tools and diagnostics [5]. On this basis, medicinal chemistry efforts have been focused on the development of compounds able to bind P2XRs with high affinity and potency, to be used as pharmacological tools for the characterisation of the receptor roles and functions, as well as therapeutic tools. A special focus was on identifying molecules suitable for their labelling as radioligands, firstly for the marking and mapping of P2XRs throughout the body, and then for in vitro radioligand binding assay studies aimed at evaluating novel potential P2XR ligands. Furthermore, given the increasing amount of evidence indicating key physiological roles of receptors like P2X7R and P2X4R in the central nervous system (CNS) and the effect of their altered expression in neurodegenerative or neuroinflammation-related disorders, P2X7R and P2X4R have progressively become potential biomarkers for CNS inflammatory/pathological conditions [6,7,8,9,10,11]. This led to several studies aimed at developing radiolabelled ligands with suitable pharmacokinetic properties to cross the blood–brain barrier (BBB) and label these proteins, with the potential to be used as diagnostic tools alone or in combination with currently standard PET imaging tools. Studies in vivo in humans have already been performed and show a good potential for clinical application of some P2XR ligands.

## 2. ATP and ATP Analogues as P2XR Radioligands

**Figure 2:**
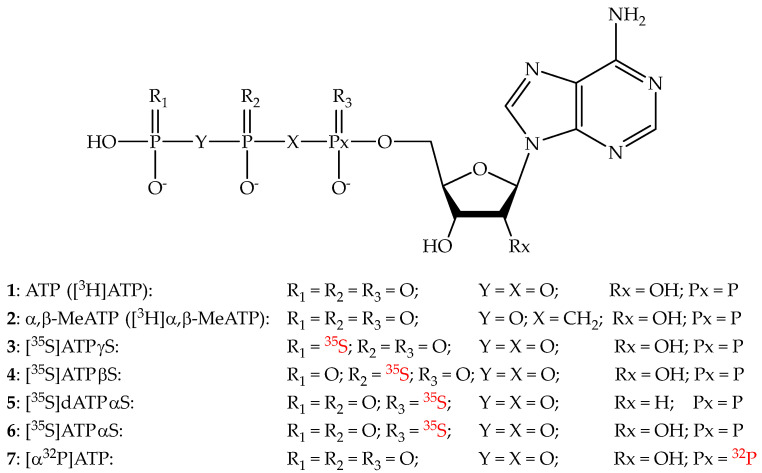
ATP and ATP analogues as radioligands.

Early studies aimed at labelling purinergic signalling players in tissue preparations were performed by using the endogenous ligand ATP in a radioactive form (Figure 2). Studies reported in the early 1980s described the use of [^3^H]ATP (**1**) as a tool to label ATP binding sites in membrane preparations of rabbit urinary bladder [12,13]. Since ATP is a compound easily subjected to hydrolysis, the authors of this study conducted their experiments at 0 °C. Further experiments reported by Yegutkin and Burnstock demonstrated the possibility of performing P2 receptor labelling and radioligand binding assays with [^3^H]ATP, but under complete inhibition of ecto-ATPase activity [14].

The development of ATP analogues like alpha,beta-methyleneATP and beta,gamma-methyleneATP (α,β-MeATP and β,γ-MeATP, respectively), as well as sulphur-containing ATP analogues ATPalphaS, ATPbetaS, and ATPgammaS (ATPαS, ATPβS, and ATPγS, respectively), led to compounds with higher chemical stability and less susceptible to ATPase activity, hence suitable for purinergic receptor labelling in various preparations (Figure 2). These compounds, together with their radioactive analogues, were of critical importance for labelling P2XRs in various tissues of the body and for detecting the P2XR subtypes.

The pharmacological tool α,β-MeATP is a typical standard for experiments involving P2XRs, given its potent agonist profile and its ability to induce receptor desensitisation. Its radioactive analogue [^3^H]α,β-MeATP (**2**) was largely employed for labelling experiments of P2XRs in tissue preparations. Following the above-cited experiments made with [^3^H]ATP, several studies of P2XR labelling and radioligand binding experiments at bladder preparations with [^3^H]α,β-MeATP were reported, leading also to the early characterisation of P2XR ligand affinity. Burnstock, Bo, and colleagues performed the first P2XR labelling studies with bladders using the same tool, also demonstrating species differences in P2X-purinoceptor densities in the urinary bladders of rats, guinea pigs, and rabbits [15,16]. They also characterised [^3^H]α,β-MeATP binding sites in human and cat bladder preparations, consistent with the presence of P2XRs [17,18]. Radioligand binding studies performed at bladder preparations with [^3^H]α,β-MeATP provided the first ranking of P2XR ligands based on their binding affinity for these receptors (even if without the identification of a specific P2XR subtype involved) [19], as well as indications for structure-activity relationships [20,21] and characterisation of the competitive antagonism profile of the pyridoxal phosphate derivative PPADS [22]. In the same years, P2XR labelling experiments with [^3^H]α,β-MeATP were also performed on various other peripheral and brain tissues [23,24]. At the rat vas deferens, [^3^H]α,β-MeATP radioligand binding experiments were conducted using various cation concentrations and with P2XR antagonists [25,26,27]. At the same tissue, binding sites for [^3^H]α,β-MeATP and [^35^S]ATPγS (**3**) were identified (in agreement with what was observed in the human bladder [28]) and it was also observed that various P2XR ligands presented different profiles in displacing the two radioligands [29]. [^3^H]α,β-MeATP was also used to label and characterise ATP binding sites and then P2XRs in vessel preparations (from aortic endothelium to pulmonary/umbilical vessels to cerebral arteries) [30,31,32,33,34]. [^3^H]α,β-MeATP, [^35^S]ATPβS (**4**), and [^35^S]dATPαS (**5**) were also used to label and characterise ATP binding sites and P2XRs in the inner ear [35,36]. Labelling of binding site for [^3^H]α,β-MeATP was performed at rat brain tissues, detecting potential P2XRs in the spinal cord, medulla oblongata, cerebellar cortex, and other brain areas [37,38,39,40,41].

In some cases, the results were misleading due to the varying degrees of sensitivity of the observed “P2XR binding sites” based on the radioligand used for the experiment. With the first characterisation studies of P2XR subtypes by using [^3^H]α,β-MeATP, [^35^S]ATPαS (**6**), and [^35^S]ATPγS and the cloning of each P2XR subtype [42,43,44,45,46,47], it was possible also to characterise the potency and selectivity of ATP itself and its analogues for the various members of this receptor family. This also led to the identification of the most suitable ATP-analogue to be used as a pharmacological tool for a given P2XR subtype and, in some cases, also for specific P2XR heterotrimers. It was observed that α,β-MeATP has a high nanomolar–low micromolar potency at the P2X1R, P2X3R, and P2X3/2R, and it is basically inactive at the P2X2R, while its analogue β,γ-MeATP is a potent P2X1R agonist, with the potency at the P2X3R being much lower. Hence, ATP-analogue radioligands were then chosen based on the P2XR subtype expressed on the analysed cell line or on the subtype representing the highest population or the target of interest in a given tissue. In any case, the lack of a clear pharmacological selectivity for specific P2XR subtypes of ATP-based analogues (in particular at the P2X7R, which generally presents low affinity for ATP analogs) and their ability to bind also non-P2XR targets (even not belonging to the purinergic signalling system) have represented two bottlenecks for the development of ATP analogue radioligands of wide utility for studies at the P2XRs [48]. [^3^H]α,β-MeATP was still used for some pharmacological studies involving the bladder [49,50,51,52,53,54] and for binding and thermodynamic characterisation of various ligands at P2X1R and P2X3R [55], while [α-^32^P]ATP (**7**) was used to characterise the non-competitive binding mode of the P2XR antagonist Suramin at the P2X2R [56]. Among the ATP-based P2XR ligands, the ATP competitive antagonist TNP-ATP can be mentioned not as a basis for the development of a radioligand, but since its trinitrophenyl moiety provides fluorescence to the molecule, it may be used as a probe. On the other hand, despite the high affinity for some P2XR subtypes [57,58], to our knowledge, no study has been reported with the use of TNP-ATP as a fluorescent probe to specifically label P2XRs in cell or tissue preparations [59].

## 3. ATP Competitive Non-Nucleotide P2XR Radioligands

**Figure 3:**
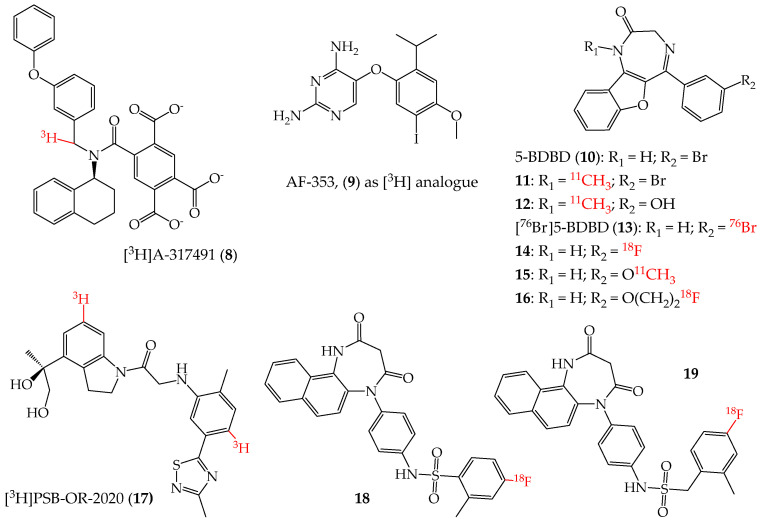
Non-nucleotide radioligands of P2X3R and P2X4R (protonation state at pH = 7.4).

To date, a limited number of ATP-competitive non-nucleotide molecules have been reported as P2XR modulators. Among them, the compound A-317491 was developed by Abbott as an inhibitor of the human and rat P2X3R and P2X2/3R. Interestingly, its potency is higher at the P2X2/3R heterotrimer than the P2X3R homotrimer in human (IC_50_ = 9 nM and 22 nM, respectively), while in rat, an opposite profile is observed (IC_50_ = 92 nM and 22 nM in P2X2/3R and P2X3R, respectively) [60]. Several works have been reported that focus mainly on the role of P2X3R in nociception and with the use of A-317491 as a pharmacological tool [61,62,63,64]. An X-ray structure of the human P2X3R in complex with A-317491 was also reported, providing a detailed description of the ligand–target interaction at the orthosteric binding site [65]. As a tritiated analogue, [^3^H]A-317491 (**8**, Figure 3) was shown to bind P2X3R and P2X3/2R with nanomolar and sub-nanomolar dissociation constants (K_d_ = 8.4 nM and 0.9 nM, respectively) [66]. Although endowed with good affinity and selectivity for these receptors, the use of [^3^H]A-317491 as a radioligand tool was reported only in a few works [67,68]. Anyway, it is a useful tool for labelling the P2X3R and P2X2/3R and for pharmacological evaluation through radioligand binding studies of novel potential orthosteric ligands of the same receptors.

## 4. Non-Competitive P2XR Radioligands

**Figure 4:**
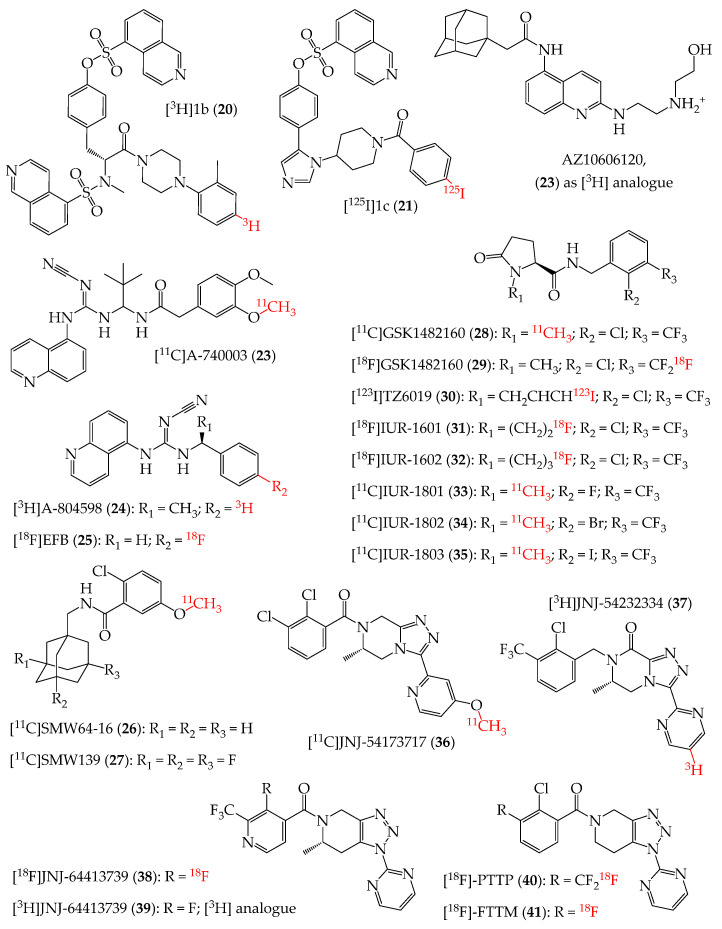
Radioligands of P2X7R (protonation state at pH = 7.4).

Given the high degree of conservation of the ATP binding site, only a few ATP derivatives endowed with some P2XR subtype selectivity have been synthesised and reported to date. On the other hand, several classes of non-competitive (allosteric) modulators of the P2XRs have been developed (Figure 3 and Figure 4) [5]. The great majority of these compounds are P2X7R modulators, although some key compounds active at other P2XR subtypes have been reported in recent years.

### 4.1. P2X3R Allosteric Modulators as Radioligands

The P2X3R receptor has been largely studied for its roles in nociceptive signal transmission and as a possible target for the development of novel analgesic compounds. To date, compounds targeting this receptor have been approved or have been studied in clinical trials mainly as tools to treat some forms of chronic cough.

Screening efforts by Roche led to the identification of diaminopyrimidine-based analogues of the antibacterial drug trimethoprim as P2XR ligands [69,70]. The first compound reported in this series, RO-3, showed high nanomolar/low micromolar inhibitory potency at the P2X3R and P2X2/3R [71]. Further development of this series led to the identification of the compound AF-353 (originally named RO-4), which showed binding affinity and inhibitory potency at the nanomolar range for human and rat P2X3Rs (IC_50_ = 8.7 nM and 8.9 nM at human and rat P2X3R, respectively) and for the human P2X2/3R [72]. A tritiated form of this molecule, [^3^H]AF-353 (**9**, Figure 3), was developed to estimate the binding affinity at these receptors (K_d_ = 15 nM and 14 nM at human and rat P2X3R, respectively). The results of in vivo characterisation studies showed that AF-353 is endowed with high oral bioavailability and CNS penetration. Another compound in this series, RO-4926219 (also known as AF-219 and MK-7264, later renamed as gefapixant), which is endowed with nanomolar potency at the P2X3R and P2X2/3R, was recently approved for market in Japan and then in Europe for the treatment of refractory or unexplained chronic cough [73]. In summary, current P2X3R-targeting radiolabelled compounds are useful for in vitro and ex vivo experiments; however, no P2X3R radioligand has been reported yet with a suitable profile for use as an in vivo PET imaging tool.

### 4.2. P2X4R Allosteric Modulators as Radioligands

The P2X4R is of increasing interest for its physiological roles in both CNS and peripheral tissues, and in neuroinflammation, CNS-related diseases, and cancer [11]. Radioligands of this receptor (Figure 3) have been developed for their utility as pharmacological tools in radioligand binding studies and as potential PET tracers, as recently reviewed by Federico [74]. A classical P2X4R inhibitor, 5-BDBD (**10**, Figure 3, IC_50_ at high nanomolar levels in human and rat P2X4R [75,76]), was modified to obtain ^11^C- and ^18^F-labelled analogues [77]. The compound was modified by insertion of a [^11^C]methyl group in the 1-position and by the combination of this modification with the substitution of the bromine atom in the 3-position of the 5-phenyl ring with a hydroxyl function (**11** and **12**, respectively, Figure 3). A second route of 5-BDBD modification led to the substitution of its bromine atom in the 3-position of the 5-phenyl ring with a [^76^Br]bromine or [^18^F]fluorine atom or a [^11^C]methoxy or [^18^F]fluoroethoxy group (**13–16**, respectively, Figure 3). Radioligand binding studies of these molecules were performed on membranes containing P2X4Rs, by monitoring the displacement of [^35^S]ATPγS radioligand (hence considering the analysed molecules as potential ATP competitive agents). The results of these experiments showed that the analysed compounds had no ability to displace the reference radioligand. On the other hand, later studies have shown that 5-BDBD works as an allosteric modulator of the P2X4R [75].

Two recent articles described the development of radioligands for P2X4Rs. In the first work, a dihydroindolylcarboxymethylaniline derivative (PSB-OR-2020) showed a very high antagonist activity at the human P2X4R (IC_50_ = 6.32 nM), with high selectivity versus the other P2XR subtypes [78]. This molecule was then tritiated at two sites to obtain [^3^H]PSB-OR-2020 (**17**, Figure 3), endowed with a K_d_ at the nanomolar level (K_d_ = 20.4 nM, analogous to the IC_50_ data). Radioligand binding experiments showed that its binding at the P2X4R probably occurs at an allosteric site different from the one(s) of other key P2X4R inhibitors like 5-BDBD, BAY-1797, and BX430.

In another work, P2X4R inhibitors based on the naphthodiazepinedione core were developed and tested as potential PET tracers [79]. Drug metabolism and pharmacokinetics (DMPK) parameters were also obtained to evaluate the possibility of further developing the compounds for in vivo applications. Several compounds with nanomolar/micromolar inhibitory potency at the P2X4R were obtained. Two of these compounds were then developed as [^18^F] analogues (**18** and **19**, respectively, Figure 3; IC_50_ = 64 nM and 8 nM at human P2X4R, respectively). In vitro analysis of these two molecules showed that they present high plasma protein binding. Furthermore, **18** is also endowed with high metabolic stability (mouse liver microsome test), while **19**, modified with a methylene spacer, presents lower stability. Despite their high lipophilicity, these two molecules do not appear to significantly pass the BBB and therefore do not reach the concentrations needed for PET imaging within the CNS.

In summary, medicinal chemistry efforts have led to the development of interesting P2X4R-targeting radioligands, although they do not appear to possess suitable pharmacodynamic and/or pharmacokinetic profiles to be used as in vivo radiotracers in PET imaging analyses.

### 4.3. P2X7R Allosteric Modulators as Radioligands

The P2X7R is the most studied subtype of the P2XR family due to its physiological and pathological roles, as well as its potential as a target for the development of novel pharmacological tools for cancer, neurological and neurodegenerative disorders, and inflammation-related conditions. A relevant number of studies have been reported to date describing the development of radiolabelled compounds targeting this receptor (Figure 4, Table 1), which can be used for in vitro or ex vivo radioligand binding studies and as tracers in in vivo PET imaging experiments [6,7,8,10].

In 2004, the development of an analogue of the reference P2X7R inhibitor KN-62 was reported. It showed nanomolar potency at the human P2X7R [80] and also a low nanomolar K_d_ at the same receptor when tested in its tritiated form ([^3^H]1b, **20**, Figure 4). More recently, another KN-62 analogue was labelled by the insertion of a [^125^I]iodine atom [81] to obtain a radioligand ([^125^I]1c, **21**, Figure 4), which also showed low nanomolar K_d_ in the human P2X7R. Further characterisation of these two compounds was not reported.

In 2007, researchers from GlaxoSmithKline reported the synthesis and biological characterisation of a P2X7R ligand, named compound-17, which was then renamed AZ10606120 and is currently a key pharmacological tool for studies on P2X7R. The compound is endowed with binding and potency at the human P2X7R at a low nanomolar level [82]. The tritiated form of compound-17 ([^3^H]AZ10606120, **22**, Figure 4) was used as a pharmacological tool for radioligand binding experiments in various works [83,84,85,86].

In the same years, researchers from Abbott reported the synthesis and pharmacological characterisation of compound A-740003, which is endowed with inhibitory activity at the nanomolar level against human, rat, and mouse P2X7Rs, as well as a nanomolar binding affinity at the rat receptor [87,88,89]. A few years later, an ^11^C-labelled form of this reference P2X7R inhibitor was also reported as a potential PET radiotracer for imaging of neuroinflammation [90]. [^11^C]A-740003 (**23**, Figure 4) showed a moderate metabolic stability but was not further developed as a tool for P2X7R imaging in the CNS, given its very low brain uptake.

Another key P2X7R ligand was reported in 2009 in its tritiated form, [^3^H]A-804598 (**24**, Figure 4) [88,91], bearing a cyanoguanidine moiety like A-740003 and showing binding affinity and inhibitory potency at the rat P2X7R at low nanomolar level (with analogue potency also at human and mouse receptors). Radioligand **24** was also used in ex vivo studies featuring rat brain and for radioligand binding assays for various novel P2X7R ligands [88,92,93,94,95,96,97,98,99,100,101,102]. Modification of A-804598 by the insertion of a [^18^F]fluorine atom led to the development of [^18^F]EFB (**25**, Figure 4), which showed a lower potency compared to **24** and a low brain uptake [103].

Screening and optimisation efforts led to the identification of a series of adamantanyl benzamides as promising ligands of the P2X7R [104]. Among these compounds, the derivative *N*-(adamantan-1-ylmethyl)-2-chloro-5-methoxybenzamide (later renamed as [^11^C]SMW14-D16, **26**, in its labelled form, Figure 4) showed nanomolar potency as a P2X7R inhibitor. Its optimisation by fluorination at the adamantane group led to the discovery of compound 2-chloro-5-methoxy-*N*-((3,5,7-trifluoroadamantan-1-yl)methyl)benzamide (later renamed as [^11^C]SMW139, **27**, in its labelled form, Figure 4) [105]. Pharmacological characterisation of both compounds showed that they inhibit human and mouse P2X7Rs, with nanomolar binding affinity for the human protein. Pharmacokinetic evaluation showed that SMW14-D16 has low metabolic stability, high lipophilicity, and very high plasma protein binding, while its fluorinated analogue SMW139 is endowed with an improved pharmacokinetics profile and higher metabolic stability. As radioligands, both compounds were shown to enter the brain [106,107]. Preliminary evaluation also showed no relevant toxic effects of **27** in rats [106]. Further studies were conducted with the latter compound as a potential PET tool for CNS imaging experiments in mice [108,109,110] and rats [111,112], and then in humans, with a special focus on multiple sclerosis (MS) [113,114]. The compound also entered a clinical trial for MS imaging [115].

In 2010, researchers from GlaxoSmithKline reported a series of pyroglutamic acid amide derivatives as P2X7R antagonists. One of these molecules, later renamed as GSK1482160, showed low nanomolar affinity and potency at the human P2X7R. A preliminary characterisation of its pharmacokinetics profile was also reported, showing a good safety profile in in vivo rat models [116]. The effects of single escalating doses of GSK1482160 on healthy humans were evaluated in a Phase 1 trial [117]. In a work published a few years later, it was reported that the required P2X7R inhibition in vivo in human (able to achieve >90% inhibition of Il-1β release throughout the entire dosing interval) could be reached only at doses that did not maintain a sufficient safety margin [118]. GSK1482160 was then selected to be labelled to obtain radioligands. [^11^C]GSK1482160 (**28**, Figure 4, K_d_ at a low nanomolar level for human P2X7) was studied at both preclinical and clinical levels [119,120,121]. In a preclinical model of lipopolysaccharide (LPS)-induced neuroinflammation in mice, **28** showed an increased brain uptake compared to control animals. Its brain penetration was also confirmed with micro-PET studies with macaques [121]. In the same work, the uptake of **28** in spinal cord of rat models of experimental autoimmune encephalomyelitis (EAE) was significantly higher compared to the control animals. This led also to the correlation between the higher level of uptake, the overexpression of the P2X7R, and the activation of microglia [121]. Given the high potential of **28** to be used as a PET tracer, an HPCL-based analytical method was also developed and validated to verify the conformance of **28** radiopharmaceutical preparation with purity and quality requirements for administration to human subjects, as defined by the U.S. Pharmacopeia [122]. Further characterisation of **28** was also made in healthy humans as a potential PET tracer. Despite the low brain uptake, it appears suitable for administration in PET studies aimed at monitoring P2X7R expression [123]. Very recently, a radiolabelling study on GSK1482160 led to the development of a second radioligand, [^18^F]GSK1482160 (**29**, Figure 4) [124], which showed metabolic stability after 60 min in rats (87%, 72%, and 56% intact molecule in brain, blood, and liver, respectively) and brain permeability. Administration of **29** in ankylosing spondylitis mouse models allowed for the detection of higher levels of P2X7R expression in ankle synovium and spinal endplate compared to control animals [125]. Further evaluation showed a significantly higher uptake of **29** in Alzheimer’s disease (AD) and primary tauopathy mice compared to that observed in control animals [126,127]. Its brain uptake was also analysed in rhesus macaques, demonstrating a potential for PET applications in humans [126]. Afterwards, **29** was used in a PET imaging experiment to map P2X7Rs in human glioma patients, in comparison with [^11^C]-methionine ([^11^C]-MET, a widely used amino acid tracer for radiolabelling brain tumours) [128]. High uptake of both tracers was observed in both higher-grade and contrast-enhanced gliomas, albeit with different distributions. The results suggested that **29** can be helpful to detect and analyse glioma immune microenvironment and tumour-induced neuroinflammation, and that PET analyses made with this tracer could be performed in parallel to analogue analyses made with standard PET radioligands, providing complementary information [128]. GSK1482160 was also modified to obtain analogues that could be labelled as radioligands. The substitution of its 1-methyl group with a 3-iodoallyl chain led to the development of compound TZ6019. In its radiolabelled form, [^123^I]TZ6019 (**30**, Figure 4) was shown to bind to human P2X7R at low nanomolar concentration [129]. Its potential as a radioligand was characterised only in vitro in tauopathy P301S transgenic mice, where it was helpful to detect an higher expression of P2X7R in hippocampus, cortex, and thalamus tissues, compared to the control animals [130]. In the same work describing the development of radioligand **30** [130], it was reported also the synthesis and a preliminary evaluation of another GSK1482160 analogue, TZ5038, which bears a 2-fluoroethyl chain replacing the 1-methyl group of GSK1482160. TZ5038 was then radiolabelled with [^18^F]fluorine to obtain [^18^F]IUR-1601 (**31**, Figure 4) [131,132], endowed with low nanomolar affinity at the human P2X7. Analogously to other GSK1482160-based radioligands, characterisation in in vivo models showed higher uptake of **31** in LPS-treated and AD rats compared to the control animals, and brain penetration in non-human primates. On the other hands, the metabolic rate of **31** resulted high in particular in blood and liver [132]. Its analogue [^18^F]IUR-1602 (**32**, Figure 4, presenting in the 1-position a 3-fluoropropyl chain instead of the 2-fluoroethyl chain of **31**) was synthesised and in vitro evaluated, showing a slightly lower binding affinity for the P2X7R compared to GSK1482160 and IUR-1601. This prevented further characterization of the compound in ex vivo or in vivo models as a radioligand [133]. GSK1482160 was also modified at its phenyl ring, with the substitution of the chlorine atom with fluorine, bromine, and iodine and the [^11^C] radiolabelling of these derivatives, to obtain [^11^C]IUR-1801 (**33**, Figure 4), [^11^C]IUR-1802 (**34**, Figure 4), and [^11^C]IUR-1803 (**35**, Figure 4), respectively [120]. The new derivatives showed nanomolar (**33**) and low nanomolar (**34** and **35**) binding affinity for the human P2X7R. Further characterisation of these compounds has not been reported to date.

In recent years, large screening and medicinal chemistry efforts were made by Janssen researchers to develop triazolopiperazine- and triazolopiperidine-based P2X7R inhibitors with potential as PET imaging tools. Among these molecules, compound JNJ-54173717 showed nanomolar inhibitory potency at the human and rat P2X7Rs [95]. It was then labelled to obtain [^11^C]JNJ-54173717 (**36**, Figure 4), which showed a low nanomolar K_d_ at the rat P2X7R [134]. It also showed to be highly brain-penetrant in rat and monkey models and was used to monitor neuroinflammation and P2X7R expression in a rat model of Parkinson’s disease (PD) and in human amyotrophic lateral sclerosis (ASL) brain sections [135,136]. A pilot study that used **36** to evaluate its biodistribution and ability to quantify brain P2X7Rs in human PD patients compared to healthy volunteers showed no significant differences in radioligand uptake in the two groups [137]. Radioligand **36** was the first brain-penetrant ^11^C-labeled radioligand used for quantification of P2X7R expression in the brain. The tritiated form of the compound JNJ-54232334 ([^3^H]JNJ-54232334, **37**, Figure 4) showed very high binding affinity and potency at the P2X7R, with a K_d_ of 4.9 nM at rat brain homogenate [96]. For the characterisation of the latter compound, **24** was selected as a reference tool. The results showed that **37** is not able to completely displace **24**, suggesting that the latter radioligand could bind to additional brain targets other than P2X7R. This demonstrates a higher specific binding of **37** to P2X7R [96].

**Table 1:** Overview of P2X7R-targeting radioligands.

Cpd	K_d_ (nM)	K_i_ (nM)	IC_50_ (nM) Ca^2+^	IC_50_ (nM) Pore	LogP ^a^	BBB Perm ^b^	Brain Uptake ^c^	LogVDss ^d^	Fract. unb. ^e^	CYP450 Substrate ^f^	*t*_1/2_ Radionucl.	Refs
[^3^H]1b (**20**)	3.46 (h)	-	15 (h)	-	5.43	−1.304	-	−0.463	0.349	*	12.3 y	[80]
[^125^I]1c (**21**)	1.68 (h)	-	-	0.25 (h)	5.95	−0.975	*	−0.061	0.136	*	59 d	[81]
[^3^H]AZ10606120 (**22**)	1.4 (h) 19 (r)	-	-	10 (h)	2.74	−0.515	**	0.835	0.238	*	12.3 y	[82,84]
[^11^C]A-740003 (**23**)	-	26.9–37.5 (r)	40 (h) 18–72.3 (r) 269 (m)	93 (h) 138 (r) 724 (m)	3.82	−1.378	-	0.954	0.11	*	0.3 h	[88,89,90]
[^3^H]A-804598 (**24**)	2.4–3.1 (r)	7.59 (r)	10.9 (h) 9.9–28.7 (r) 8.9 (m)	8.1 (h)	3.83	0.223	*	1.064	0.053	*	12.3 y	[88,91]
[^18^F]EFB (**25**)	-	-	1820 (h) 7244 (r) 6026 (m)	-	3.41	−0.117	*	1.465	0.083	*	1.8 h	[103]
[^11^C]SMW64-D16 (**26**)	-	8.5 (h)	25.7 (h) 1905 (m)	10.5 (h)	4.29	0.273	****	0.073	0	*	0.3 h	[105]
[^11^C]SMW139 (**27**)	-	32 (h)	24.5 (h) 158 (m)	33.9 (h)	4.57	0.42	***	0.269	0.164	*	0.3 h	[105]
[^11^C]GSK1482160 (**28**)	1.15–5.1 (h)	2.6–68 (h)	-	2.1–3.2 (h) 251 (r)	2.59	0.263	***	0.149	0.369	-	0.3 h	[101,116,118,119,120,121,123,130]
[^18^F]GSK1482160 (**29**)	4.3 (h)	2.6–68 (h)	-	2.1–3.2 (h) 251 (r)	2.59	0.263	***	0.149	0.369	-	1.8 h	[101,116,118,119,120,121,123,124,130,133]
[^123^I]TZ6019 (**30**)	19.3 (h)	6.3 (h)	-	9.7 (h)	3.91	0.131	***	0.046	0.186	*	13 h	[130]
[^18^F]IUR-1601 (**31**)	-	3.7–4.3 (h)	-	7.9–9.9 (h)	2.93	0.249	***	0.091	0.363	*	1.8 h	[130,132,133]
[^18^F]IUR-1602 (**32**)	-	23.6 (h)	-	17.8 (h)	3.32	−0.039	***	0.088	0.298	*	1.8 h	[132,133]
[^11^C]IUR-1801 (**33**)	-	54.2 (h)	-	-	2.08	−0.036	***	−0.224	0.358	-	0.3 h	[120]
[^11^C]IUR-1802 (**34**)	-	2.5 (h)	-	-	2.70	0.261	***	0.159	0.361	-	0.3 h	[120]
[^11^C]IUR-1803 (**35**)	-	1.9 (h)	-	-	2.55	0.256	***	0.161	0.364	-	0.3 h	[120]
[^11^C]JNJ-54173717 (**36**)	-	1.6 (r)	4.2–7.7 (h) 7.6–10 (r)	-	3.70	−1.086	**	−0.254	0.084	*	0.3 h	[95,134]
[^3^H]JNJ-54232334 (**37**)	4.9 (r)	0.5 (r)	0.32 (h) 31.6 (r)	-	3.45	−1.396	**	−0.208	0.083	*	12.3 y	[96]
[^18^F]JNJ-64413739 (**38**)	-	15.9 (h) 2.7 (r)	1.0 (h) 1.9 (r)	-	2.19	−1.593	**	−0.755	0.198	*	1.8 h	[138]
[^3^H]JNJ-64413739 (**39**)	7 (h)	15.9 (h) 2.7 (r)	1.0 (h) 1.9 (r)	-	2.19	−1.593	**	−0.755	0.198	*	12.3 y	[139]
[^18^F]PTTP (**40**)	12.4 (m)	16 (h) 2.8 (r)	4.2 (h) 6.8 (r) 4.0 (m)	-	2.93	−1.318	***	−0.383	0.054	*	1.8 h	[140,141]
[^18^F]FTTM (**41**)	25.3 (h)	-	-	-	2.04	−1.077	**	−0.313	0.126	*	1.8 h	[142,143]

(**^a^**) Calculated log of octanol/water partition coefficient [144]; compound protonation state at pH = 7.4. (**^b^**) BBB permeability prediction [144]. For a given compound a logBB > 0.3 is considered to readily cross the blood–brain barrier, while molecules with logBB < −1 are poorly distributed to the brain; compound protonation state at pH = 7.4. (**^c^**) Prediction of brain uptake of the analysed compound, protonation state at pH = 7.4; each molecule was analysed with four pharmacokinetic properties predictors [144,145,146,147]; the number of * symbols indicates the number of predictors indicating brain uptake. (**^d^**) Steady state volume of distribution (VDss) prediction [144]. The predicted logarithm of VDss (LogVDss) of a given compound is given as the log L/kg. VDss is considered low if below 0.71 L/kg (LogVDss < −0.15) and high if above 2.81 L/kg (LogVDss > 0.45); within parentheses is the experimental value expressed accordingly; compound protonation state at pH = 7.4. (**^e^**) Prediction of the compound fraction unbound to plasma protein [144]. For a given compound the predicted fraction that would be unbound (not bound to serum proteins) in plasma is calculated; within parentheses is the experimental value expressed accordingly; the compound protonation state at pH = 7.4. (**^f^**) Prediction for a given molecule to be a substrate of one of the CYP450 isoforms CYP2D6 and CYP3A4; the number of * symbols indicates the number of CYP450 isoforms for which the molecule is predicted as a substrate [144]; compound protonation state at pH = 7.4.

The triazolopiperidine derivative JNJ-64413739 was reported in its [^18^F]fluorine labelled form ([^18^F]JNJ-64413739, **38**, Figure 4) [138]. It showed low nanomolar inhibitory potency at human and rat P2X7R and nanomolar affinity for the same receptors. Preclinical evaluation as a PET tracer demonstrated good metabolic stability and good brain uptake in both rats and monkeys [148]. The evaluation of **38** was then made in humans by monitoring kinetic parameters and P2X7R labelling [149,150,151,152]. Further evaluation in rodent models of neuroinflammation and epilepsy demonstrated an increased uptake of **38** in brain regions injected with LPS and in brain regions associated with temporal lobe epilepsy compared to control regions, demonstrating the potential of this pharmacological tool to label regions with overexpressed P2X7R [153,154]. The use of **38** was also helpful in demonstrating the potential of P2X7R inhibition and radiolabelling to prevent the occurrence of epilepsy and to identify the risk of developing increased brain hyperexcitability in traumatic brain injury rodent models [155]. JNJ-64413739 was also developed in a tritiated form, [^3^H]JNJ-64413739 (**39**, Figure 4), and its K_d_ was found to be at a low nanomolar level in an in vitro analysis on cortical tissue sections from human patients with treatment-resistant epilepsy [139].

A 1,2,3-triazolopiperidine derivative with nanomolar affinity and potency for the human and rat P2X7Rs was identified in 2015 and initially named as compound 12d [97]. Its ^18^F-labelling led to the radioligand [^18^F]-PTTP (**40**, Figure 4), designed to work as an inflammation and neuroinflammation marker [140,141]. The results of its evaluation showed an increased uptake in inflammatory tissues compared to cancer sites, hence suggesting its potential to mark inflammation spots. Radioligand **40** also proved to label P2X7R in epileptic rat brains. Metabolism analysis showed high stability of the tool after 1 h of injection [140,141]. A close analogue of **40** was developed and reported as [^18^F]-FTTM (**41**, Figure 4) [142,143], which was used as well to monitor neuroinflammation in a rat model of temporal lobe epilepsy, showing potential to label P2X7R expression. Another application of **41** was related to the labelling of vulnerable atherosclerotic plaques and the early detection of atherosclerotic lesions.

The labelling of atherosclerotic plaques is also the target of a recently reported ^99m^Tc-labeled nanobody radiotracer as a SPECT probe [156].

## 5. Conclusions and Perspective

The development of radioligands is of key importance to provide pharmacological tools that are exploitable in in vitro and ex vivo studies, as well as in vivo imaging techniques like PET or SPECT. The choice of the application of a radioligand is based on the kind of radiolabelling process applied to the radioligand precursor. Long half-life radioligands (i.e., ^3^H- or ^125^I-labelled radiotracers, t_1/2_ = 12.5 y and 59 d, respectively) are generally employed for in vitro or ex vivo analyses. Tritiated ligands are widely used for radioligand binding assays of novel compounds. In the case of the P2XR, tritiated analogues of ATP have been used for labelling of these proteins in tissues and were helpful also for the identification of P2XR subtypes, as well as ^35^S- and ^32^P-labelled ATP derivatives. On the other hand, the use of these ligands at the P2X7R was of limited utility given the very low affinity of this receptor for ATP and its analogues, with the exception of the Bz-ATP compound, which has high potency as a P2X7R agonist but lacks significant P2XR subtype selectivity. However, radiolabelled versions of Bz-ATP have not been reported to date. A further bottleneck for the development of subtype-selective P2XR radioligands is the high conservation of the ATP binding site among the various P2XRs.

Hence, with the discovery of the first classes of allosteric modulators of these proteins, medicinal chemistry efforts were also made to develop non-competitive radioligands with good affinity and subtype selectivity. Tritiated allosteric modulators of the P2XRs were developed specifically for the P2X3R, P2X7R, and, more recently, the P2X4R subtypes. These tools enable the in vitro pharmacological characterisation of novel ligands of these proteins and, in general, for in vitro/ex vivo studies. Given the important roles these receptors play in the CNS (i.e., in neuroinflammatory and neurodegenerative conditions) as well as in the peripheral tissues, efforts were made to develop radioligands able to selectively label specific P2XR subtypes, but also with the potential to be used for in vivo studies in humans. In this sense, the studies focused mainly on the development of P2X4R or P2X7R radiotracers, where the key factors considered in the process were interspecies receptor affinity, brain uptake, and metabolic stability.

As first, the modification of P2X4R/P2X7R ligands with good affinity/potency at both human and rodent receptors with the insertion of short half-life isotopes like a [^18^F]fluorine or an [^11^C]carbon (t_1/2_ = 1.8 h and 0.3 h, respectively) had the advantages to not highly impact the receptor affinity and to lead to compounds with suitable profile for in vivo imaging studies, with ^18^F-labeled compounds endowed with longer half-life compared to the ^11^C-labeled ones (with the insertion of a [^123^I]iodine atom, t_1/2_ = 13 h, being an alternative option). On the other hand, for a clinical application, the short half-life of these radiotracers requires a fast and efficient labelling method, which must be conducted in proximity to the site of clinical/diagnostic imaging analysis. Still, about the target affinity, the presence of a number of P2X7R variants (due to single-nucleotide polymorphisms or alternative splicing mechanisms) may affect the affinity and potency of P2X7R-targeting tools, and, in particular, the radiotracer’s ability to label the receptor. Some of these P2X7R variants are present in specific conditions (i.e., brain diseases) or are associated with altered response to agonists (gain-of-function or loss-of-function) or inability to form the prolonged activation-induced macropore [157,158]. The development of tools capable of detecting and labelling these variants could be of critical help, not only from a diagnostic point of view but also for the planning of a therapy targeting a specific P2X7R variant.

Secondly, some of these derivatives showed a good degree of brain uptake, indicating potential to be used for the detection of P2XRs and for evaluating their level of expression within the CNS. In this sense, an advantage of using brain-penetrant P2X7R-radiotracers is related to their ability to detect regions presenting overexpression of this protein, which is associated with neuroinflammation; MS, AD, ALS, and PD; glioma; and enhanced risk of developing increased brain hyperexcitability. On the other hand, radiotracers with low or no brain penetration showed high potential for detecting and imaging inflammation-related lesions or cancer, with P2X7R ligands showing higher uptake in the inflammatory region compared to cancer tissues. Therefore, both brain-permeable and non-permeable P2X7R radioligands could also be helpful in co-administration with other PET tracers, leading to improved imaging of CNS and non-CNS pathological conditions.

Third, the metabolic stability and, in general, a suitable pharmacokinetics profile of the radioligands are obvious factors. In this sense, besides BBB penetration and other general pharmacokinetic parameters, an additional aspect that must be considered is the potential formation in vivo of radiometabolites that could label non-P2XR off-targets, resulting in a misleading output of the imaging experiments.

In the P2X4R, all these goals were only partially achieved, with no radiotracer reported to date presenting a suitable profile to be used for in vivo imaging studies. Given the increasing number of medicinal chemistry efforts to develop selective P2X4R modulators and the availability of receptor structures useful for a more efficient structure-based design, P2X4R radiotracers with an improved profile could be available in the near future.

Regarding the P2X7R, a significant number of radioligands targeting this protein were developed for both CNS and peripheral tissue imaging. Some of these derivatives were also clinically evaluated in vivo in humans. These results are quite promising, making P2X7R a target for additional radiodiagnostic tools to be provided to the scientific community and to be used in combination with existing radiotracers for more comprehensive imaging studies of inflammatory, neuroinflammatory, and cancer-related conditions.

## Data Availability

No new data were created or analyzed in this study. Data sharing is not applicable to this article.

## Abbreviations

The following abbreviations are used in this manuscript:

PET	Positron emission tomography
SPECT	Single-photon emission computed tomography
P2XR	P2X receptor
AR	Adenosine receptor
CNS	Central nervous system
BBB	Blood–brain barrier
DMPK	Drug metabolism and pharmacokinetics
MS	Multiple sclerosis
LPS	Lipopolysaccharide
EAE	Experimental autoimmune encephalomyelitis
AD	Alzheimer’s disease
PD	Parkinson’s disease
ASL	Amyotrophic lateral sclerosis
